# Acute Febrile Illness Among Children in Butajira, South–Central Ethiopia During the Typhoid Fever Surveillance in Africa Program

**DOI:** 10.1093/cid/ciz620

**Published:** 2019-10-30

**Authors:** Mekonnen Teferi, Mulualem Desta, Biruk Yeshitela, Tigist Beyene, Ligia Maria Cruz Espinoza, Justin Im, Hyon Jin Jeon, Jong-Hoon Kim, Frank Konings, Soo Young Kwon, Gi Deok Pak, Jin Kyung Park, Se Eun Park, Melaku Yedenekachew, Jerome Kim, Stephen Baker, Won Seok Sir, Florian Marks, Abraham Aseffa, Ursula Panzner

**Affiliations:** 1 Armauer Hansen Research Institute, Ministry of Health, Addis Ababa, Ethiopia; 2 International Vaccine Institute, Seoul, South Korea; 3 Technology and Innovation Institute, Addis Ababa, Ethiopia; 4 Graduate School of Public Health, Yonsei University, Seoul, South Korea; 5 Hospital for Tropical Diseases, Welcome Trust Major Overseas Program, Oxford University Clinical Research Unit, Ho Chi Minh City, Vietnam; 6 Department of Medicine, University of Cambridge, United Kingdom

**Keywords:** children, acute febrile illness, Typhoid Fever Surveillance in Africa Program (TSAP), Butajira, Ethiopia

## Abstract

**Background:**

Clearly differentiating causes of fever is challenging where diagnostic capacities are limited, resulting in poor patient management. We investigated acute febrile illness in children aged ≤15 years enrolled at healthcare facilities in Butajira, Ethiopia, during January 2012 to January 2014 for the Typhoid Fever Surveillance in Africa Program.

**Methods:**

Blood culture, malaria microscopy, and blood analyses followed by microbiological, biochemical, and antimicrobial susceptibility testing of isolates were performed. We applied a retrospectively developed scheme to classify children as malaria or acute respiratory, gastrointestinal or urinary tract infection, or other febrile infections and syndromes. Incidence rates per 100 000 population derived from the classification scheme and multivariate logistic regression to determine fever predictors were performed.

**Results:**

We rarely observed stunting (4/513, 0.8%), underweight (1/513, 0.2%), wasting (1/513, 0.2%), and hospitalization (21/513, 4.1%) among 513 children with mild transient fever and a mean disease severity score of 12 (95% confidence interval [CI], 11–13). Blood cultures yielded 1.6% (8/513) growth of pathogenic agents; microscopy detected 13.5% (69/513) malaria with 20 611/µL blood (95% CI, 15 352–25 870) mean parasite density. Incidences were generally higher in children aged ≤5 years than >5 to ≤15 years; annual incidences in young children were 301.3 (95% CI, 269.2–337.2) for malaria and 1860.1 (95% CI, 1778.0–1946.0) for acute respiratory and 379.9 (95% CI, 343.6–420.0) for gastrointestinal tract infections.

**Conclusions:**

We could not detect the etiological agents in all febrile children. Our findings may prompt further investigations and the reconsideration of policies and frameworks for the management of acute febrile illness.

Febrile illnesses are a public health challenge, especially in developing countries, particularly for health professionals who often have to rely on empirical clinical diagnoses due to limited diagnostic capacity. However, empirical diagnoses may lead to poor patient management, which can result in disease recurrence, deterioration, and potentially death. Importantly, the inappropriate use of antimicrobials may adversely affect the gut microbiome and facilitate the development of resistance to antimicrobial drugs [1].

According to the Ethiopian Ministry of Health, respiratory and gastrointestinal tract infections were the leading causes of morbidity among children in Ethiopia in 2014 and 2015 [2, 3]. Children suffered primarily from bloody/non-bloody diarrhea and upper/lower respiratory infections in addition to malaria, skin and eye infections, and helminthiases. Common causes of death were acute respiratory tract infections, diarrhea, malaria, human immunodeficiency virus/AIDS, meningitis and measles, noncommunicable perinatal complications (birth asphyxia, prematurity, neonatal sepsis), and severe malnutrition [2–4].

The Typhoid Surveillance in Africa Program (TSAP) assessed the incidence and etiological agents of acute febrile illness in children aged ≤15 years recruited in Butajira, south–central Ethiopia from January 2012 to January 2014. TSAP was established in 2009 to conduct standardized prospective blood culture–based fever surveillance at healthcare facilities at 13 sites in the following 10 sub-Saharan African countries: Burkina Faso, Ethiopia, Ghana, Guinea-Bissau, Kenya, Madagascar, Senegal, South Africa, Sudan, and Tanzania [5]. The intent of the program was to generate the evidence needed to support policy makers in the introduction of preventive measures, particularly of safe and effective vaccines for typhoid fever and invasive nontyphoidal *Salmonella* diseases.

We have noted low positivity rates for blood cultures from Butajira in contrast to other TSAP sites. Therefore, in addition to analyzing prospectively collected surveillance data, we developed a retrospective case classification scheme guided by unpublished in-country charts and published literature while making the best use of clinical and laboratory surveillance data.

## METHODS

### Ethical Considerations

We received ethical clearance for TSAP from the ethical committees of the International Vaccine Institute, South Korea; the Armauer Hansen Research Institute/All African Leprosy, Tuberculosis and Rehabilitation Training Center; and the Ethiopian National Research Ethics Review Committee. We obtained written informed consent or a fingerprint in case of illiteracy from parents, caretakers, or guardians of participating children aged ≤15 years [5]. Study participation was voluntary. Data from enrolled children were kept in strict confidence and made available only to coinvestigators and staff directly involved in the study.

### Study Site

**Figure 1.**
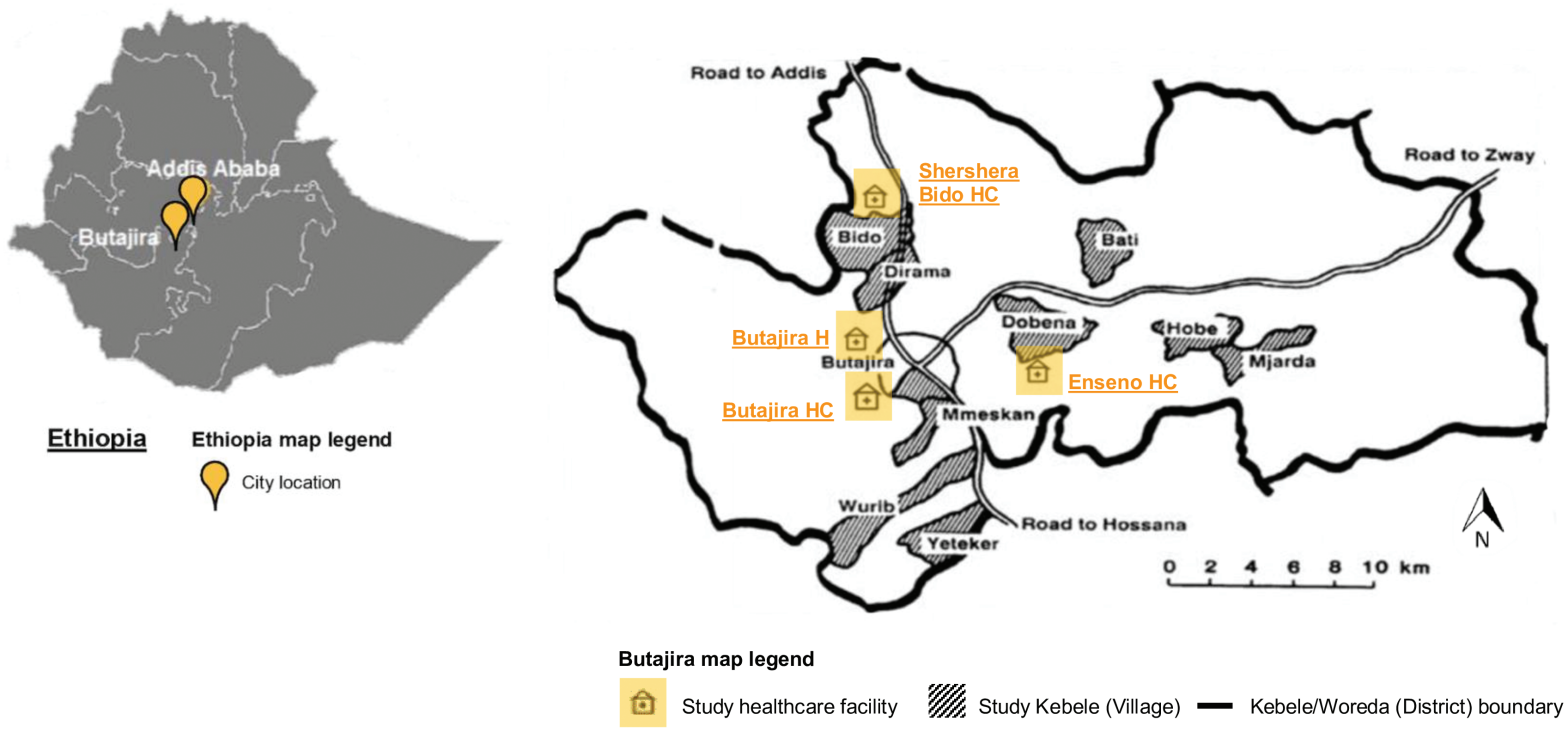
Map of Ethiopia and the study site, Butajira, Ethiopia, January 2012 to January 2014. The map on the left shows the location of the capital Addis Ababa and Butajira within Ethiopia indicated by orange flags. The map on the right illustrates the study site Butajira, including its kebeles, or villages, selected and the location of the Typhoid Fever Surveillance in Africa Program recruitment healthcare facilities. Both maps were modified from their original sources [10, 11]. Kebeles are pictured as gray-shaded areas. One urban kebele, Butajira-04 (population, 20 026), and 9 rural kebeles, Wuribe (population, 6172), Yeteker (population, 5652), Dobena-Gola (population, 5613), Hobe-Jare-Demeka (population, 5478), Bati-Lijano (population, 5464), Misrak-Meskan (population, 4159), Mekakelegna-Jare-Demeka (population, 3705), Dirama (population, 3118), and Shershera-Bido (population, 2578), comprised the site; kebeles were classified as urban and rural as known locally. A slightly different kebele name may be used depending on the data source (eg, Butajira-04 or Butajira, Dobena-Gola or Dobena, Hobe-Jare-Demeka or Hobe, Bati-Lijano or Bati, Misrak-Meskan or Mmeskan, Mekakelegna-Jare-Demeka or Mjarda, Wuribe or Wurib, Shershera-Bido or Bido). The location of each recruitment healthcare facility is indicated as an orange building symbol. Abbreviations: H, hospital; HC, health center.

Ethiopia is comprised of 9 regional states and 2 chartered cities that are further subdivided into zones, subcities, woredas (district), and kebeles (village). Butajira is located at the base of the Zebidar massif in the Gurage Zone of the Southern Nations, Nationalities and Peoples’ Region approximately 130 km from Addis Ababa (Figure 1). This region has arid, dry lowlands at about 1500 m altitude and cool mountainous areas up to 3500 m altitude; the main wet season is from June to September, with an average annual rainfall of 945 mm [6]. Butajira has 86 kebeles of which 1 urban and 9 rural kebeles with a combined population of 61 965 in 2012 were selected as study site (Figure 1) [7]. The population is largely characterized by subsistence farmers of low literacy and high poverty [8, 9].

### Prospective Fever Surveillance

All febrile in- and outpatients aged ≤15 years who sought healthcare at one of the primary recruitment healthcare facilities, Butajira Health Center, Shershera Bido Health Center, and Enseno Health Center, or the secondary healthcare facility, Butajira Hospital, were approached for participation if they met our inclusion criteria (Table 1) [5]. All healthcare facilities are public and provide general medical care to patients. Upon recruitment, trained study staff took the clinical history, performed physical examinations, and collected patient data including age; sex; weight and height; place of residence; clinical symptoms; fever characteristics; pretreatment with antimalarials, analgesics, or antibacterials; contact with a febrile person; travel outside the study area; physician’s presumptive diagnosis; and antimicrobial drugs prescribed.

**Table 1.** Eligibility Criteria of Children for Participation, Butajira, Ethiopia, January 2012 to January 2014

Eligible Pediatric Inpatient	Fever history within past 72 hours or temperature ≥38.0°C (tympanic) or ≥37.0°C (axillary)	Parent(s)/ caretakers(s)/ guardian(s) of child consented	Child resided in study area
Eligible Pediatric Outpatient	Temperature ≥38.0°C (tympanic) or ≥37.0°C (axillary)	Parent(s)/ caretakers(s)/ guardian(s) of child consented	Child resided in study area

Study staff collected 1–3 mL of whole blood by sterile syringe from each child, adhering to safety procedures for handling human infectious materials. Blood was inoculated in a single aerobic blood culture bottle (Bactec PedsPlus Medium, Becton Dickinson, NJ) and incubated in an automated, continuously monitored blood culture instrument (Bactec 9050, Becton Dickinson, NJ). The syringe graduation was observed during bottle inoculation to avoid under- and overfilling. Single malaria microscopy from thin and thick smears and a complete blood count (CBC) were performed if the blood volume collected was sufficient. Of cultures that yielded growth, common microbiological and biochemical identification, for example, Gram stain, subcultures on selective and nonselective media, catalase, oxidase, streptococcal grouping (Thermo Fisher Scientific, Waltham, MA), API20E, or APINH (Analytical Profile Index for the identification and differentiation of *Enterobacteriaceae*, *Neisseria*, and *Haemophilus*; BioMérieux, Marcy l’Etoile, France), and O-, H-, and Vi-serotyping of *Salmonella* (Sifin Diagnostics, Berlin, Germany) was performed. We classified bacteria isolated as probable contaminant, contaminant, or pathogen based on the literature and in consultation with microbiologists [5, 12]. All organisms were subjected to antimicrobial susceptibility testing using the Kirby-Bauer disk diffusion method according to Clinical Laboratory and Standards Institute guidelines [13]. Study staff collected the following laboratory data: blood culture data including time point of blood collection, blood volume inoculated, and start/end of culture incubation, malaria microscopy inclusive of parasite count and species, white blood cells (WBCs), red blood cells (RBCs) and platelets, and antimicrobial susceptibility testing (AST). Internal quality control procedures utilizing reference strains of the American Type Culture Collection were implemented [5]. Study staff communicated findings of all investigations to the care team upon availability for prompt treatment in accordance with Ethiopian national guidelines. All examinations were performed following standard operating procedures developed for TSAP.

### Retrospective Case Classification Scheme

We developed an algorithm due to sparse blood culture growth outcomes guided by unpublished charts and logbooks from healthcare facilities in Ethiopia and published literature in order to classify pediatric participants as having malaria, acute respiratory tract infection (ARTI), gastrointestinal infection (GI), urinary tract infection (UTI), or other febrile infections and syndromes (OFIS); details are outlined in Table 2 [14–26]. The following data derived from prospective fever surveillance were used: age; sex; fever characteristics inclusive of body temperature; appearance and duration; symptoms including diarrhea, headache, constipation, sore throat, rash, cough, vomiting, and abdominal pain; physicians’ presumptive diagnoses; and results of malaria microscopy, CBC counts, and blood culture. Two scientists on our team independently performed the actual classification. Their results were compared by a third independent scientist, and consensus on the final classification was sought in case of discrepancies.

**Table 2.** Case Classification Scheme for Participating Children, Butajira, Ethiopia, January 2012 to January 2014

Case Classification	Required Clinical Criteria	Supportive Clinical Criteria	Supportive Laboratory Criteria
Malaria	All presentations	Temperature ≥40.0°C *or* anemia (severe)	Malaria microscopy positive *or* low platelet count *or* low white blood cell count
Acute respiratory tract infection	All presentations *and* cough	Temperature ≥38.0°C *or* fast breathing *or* difficult breathing *or* sore throat	Blood culture positivity *or* high white blood cell count
Gastrointestinal infection	All presentations *and* abdominal complications	Diarrhea *or* vomiting *or* fever for ≥3 days *or* headache *or* loss of appetite *or* rash *or* cough	Blood culture positivity *or* low platelet count *or* normal/low white blood cell count
Urinary tract infection	All presentations	Temperature 40.0°C–41.0°C *or* fever for ≥3days *or* female *or* young age *or* low body weight *or* abdominal complications	Low platelet count
Other febrile infections and syndromes	All presentations *and* fever	Temperature ≥38.0°C *or* fever for ≥2 days (no localized source)	…

### Data Analyses

We computed baseline results as absolute and relative frequencies. Body temperatures of ≤38.9°C, ≥39.0 to ≤39.9°C, and ≥40.0°C were categorized as mild, moderate, and high fever, respectively. We calculated the malaria parasite density as the number of parasites counted divided by the WBC counted multiplied by the patient WBC count per microliter, including its mean and 95% confidence intervals (CIs) per standard formula for a single mean [27]. The *z* scores for height-for-age or stunting, weight-for-age or underweight, and weight-for-height or wasting were generated as the difference between the patient value and the median World Health Organization/National Center for Health Statistics/Centers for Disease Control and Prevention (WHO/NCHS/CDC) reference population value divided by the WHO/NCHS/CDC reference population standard deviation [28]. We computed the mean and median physiology- and simple scale-based disease severity score of participants accompanied by 95% CIs and the interquartile range (IQR) by applying numerical weighting to each variable value and summarizing weighted individual scores [29]. The variables that were used based on availability and consensus of our research team included age; fever characteristics including body temperature and duration; hospital admission; symptoms inclusive of diarrhea, headache, constipation, sore throat, rash, cough, vomiting, and abdominal pain; *z* scores for height-for-age, weight-for-age, and weight-for-height; and RBC, WBC, and platelet counts. A weighting from 0 to 4 denotes an increasing deviation from normal with increasing scores and from 0 to 1 the presence or absence of a variable. We assessed the mean elapsed time from blood collection to culture incubation, the mean blood volume inoculated, and the mean incubation period until positivity or negativity as detected by the blood culture instrument accompanied by 95% CIs. We computed overall and stratified crude incidences by dividing the number of new cases derived from applying our case classification scheme by the number of children at risk (≤5 years: 17 634; >5 to ≤15 years: 13 817), including 95% CIs per standard formula for a rate. We generated odds ratios (ORs) with 95% CIs and multivariate logistic regression analyses to determine predictors for acute febrile illness, applying stepwise selection of variables from prospective fever surveillance and retrospective case classification. A *P* value of <.05 was considered statistically significant. All analyses were performed with the Statistical Package for the Social Sciences software (SPSS, version 20, IBM).

## RESULTS

### Baseline Characteristics

We observed nearly equal distributions of sex (male, 281/513; 54.8%), age (≤5 years, 247/513; 48.1%), setting (urban, 229/513; 44.6%), and recruitment season (dry, 219/513; 42.7%) among the 513 children recruited (Supplementary Tables 1–3). Adequate values for 504 (98.2%), 495 (96.5%), and 497 (96.9%) children for height-for-age, weight-for-age, and weight-for-height were detected, respectively; very few children were stunted, underweight, or wasted (Supplementary Figure 1). The mean and median disease severity score among all children was 12 (95% CI, 11–13) and 11 (IQR, 9–14; range, 2–29), respectively.

Of all children, 182 (35.5%) presented at Butajira Hospital and 21 (4.1%) were admitted (Supplementary Table 1). Of those admitted, 20 (95.2%) were aged ≤5 years, 11 (52.4%) had moderate fever, 14 (85.7%) presented during the wet season, 14 (66.7%) were from rural kebeles, and 17 (81.0%) and 3 (14.3%) were classified as having ARTI and GI, respectively (Supplementary Figure 2). Of all participants, 295 (57.5%) were diagnosed with headache, 252 (49.1%) with cough, 173 (33.7%) with vomiting, 115 (22.4%) with diarrhea, 340 (66.3%) with mild fever, and 469 (91.4%) with fever that lasted 3 days or longer (Supplementary Table 1). None of the children met the criteria for UTI, but 255 (49.7%) were classified as having ARTI, 132 (25.7%) as having OFIS, 69 (13.5%) as having malaria, and 57 (11.1%) as having GI (Supplementary Tables 2 and 4). Parents, caretakers, or guardians reported treatment with antimalarials, antibacterials, or analgesics; contact with a febrile person, and traveling outside of the study site 1 week prior to visiting a recruitment healthcare facility for 119 (21.4%), 40 (7.8%), and 13 (2.5%) children, respectively (Supplementary Tables 1 and 3).

### Blood Findings

CBC data revealed normal WBC counts for 140 (56.7%) children aged ≤5 years and 108 (40.6%) children aged >5 to ≤15 years. Low lymphocyte counts were found among 137 (55.5%) and 70 (26.3%) as well as elevated neutrophil counts among 117 (47.4%) and 60 (22.6%) children aged ≤5 and >5 to ≤15 years, respectively. RBC counts and associated parameters as well as platelet counts were normal among the majority of children, except for a lowered mean corpuscular hemoglobin concentration (MCHC) in 139 (56.3%) and 92 (34.6%) children aged ≤5 years and >5 to ≤15 years, respectively.

Of 69 (13.5%) children who tested positive for malaria by microscopy, 42 (60.9%) were males, 46 (66.7%) were aged >5 to ≤15 years, 38 (55.1%) had high fever, 53 (76.8%) were from rural kebeles, and 44 (63.8%) presented during the wet season; parents, caretakers, or guardians of 4 (5.8%) children recalled pretreatment with antimalarials. We diagnosed *Plasmodium falciparum* and *Plasmodium vivax* parasites in 15 (21.7%) and 54 (78.3%) children, respectively, including 16 (23.2%) slides with *P. vivax* gametocytes. The mean parasite density was 20 611/µL blood (95% CI, 15 352–25 870) with a mean of 21 881/µL blood (95% CI, 10 808–32 954) for *P. falciparum* and 19 931/µL blood (95% CI, 14 042–25 820) for *P. vivax*.

### Blood Culture Findings

Of all 513 blood cultures performed, 8 (1.6%) yielded growth for bacterial or fungal pathogens and 54 (10.5%) for contaminants; no growth was detected in 440 (85.8%) cultures. Of 54 contaminants, 13 (24.1%) were coagulase-negative *Staphylococci*, 12 (22.2%) *Bacillus* spp., 4 (7.4%) *Micrococcus* spp., and 7 (13.0%) coryneform species. The mean blood volume inoculated into culture bottles was 2.4 mL (95% CI: 2.2–2.6) for all cultures, 2.5 mL (95% CI: 2.2–2.7) for growth-negative cultures, 1.8 mL (95% CI: 0.5–3.0) for cultures with pathogens, and 2.3 mL (95% CI: 1.7–2.9) for cultures with contaminants. The mean incubation period until positivity or negativity detected by the culture instrument was 5.1 days (95% CI, 4.6–5.5) for all cultures, 5.6 days (95% CI, 5.0–6.1) for growth-negative cultures, 2.0 days (95% CI, 0.6–3.4) for cultures with pathogens, and 1.9 days (95% CI, 1.4–2.4) for cultures with contaminants.

Among 8 children with cultures positive for pathogenic organisms, 7 (87.5%) were aged ≤5 years, 7 (87.5%) were females, 6 (75.0%) had moderate to high fever, and 4 (50.5%) and 3 (37.5%) were classified as having GI and ARTI; parents, caretakers, or guardians of 2 (25.0%) children reported pretreatment with antibacterials or analgesics. Of the pathogenic agents, 3 (37.5%) were gram-negative (*Escherichia coli* [2], *Salmonella* Typhi [1]) and 4 (50.0%) were gram-positive (*Streptococcus pneumoniae* [3], *Staphylococcus aureus* [1]) bacteria; the remainder were *Candida* spp. [1]. AST revealed that pathogenic bacteria were susceptible to all antimicrobials tested, that is, amoxicillin/clavulanate, chloramphenicol, ampicillin, ciprofloxacin, ceftazidime, nalidixic acid, ceftriaxone, and trimethoprim/sulfamethoxazole, except for 1 *E. coli* isolate that exhibited resistance to ampicillin, nalidixic acid, and trimethoprim/sulfamethoxazole.

### Incidence of Infections and Syndromes

**Figure 2.**
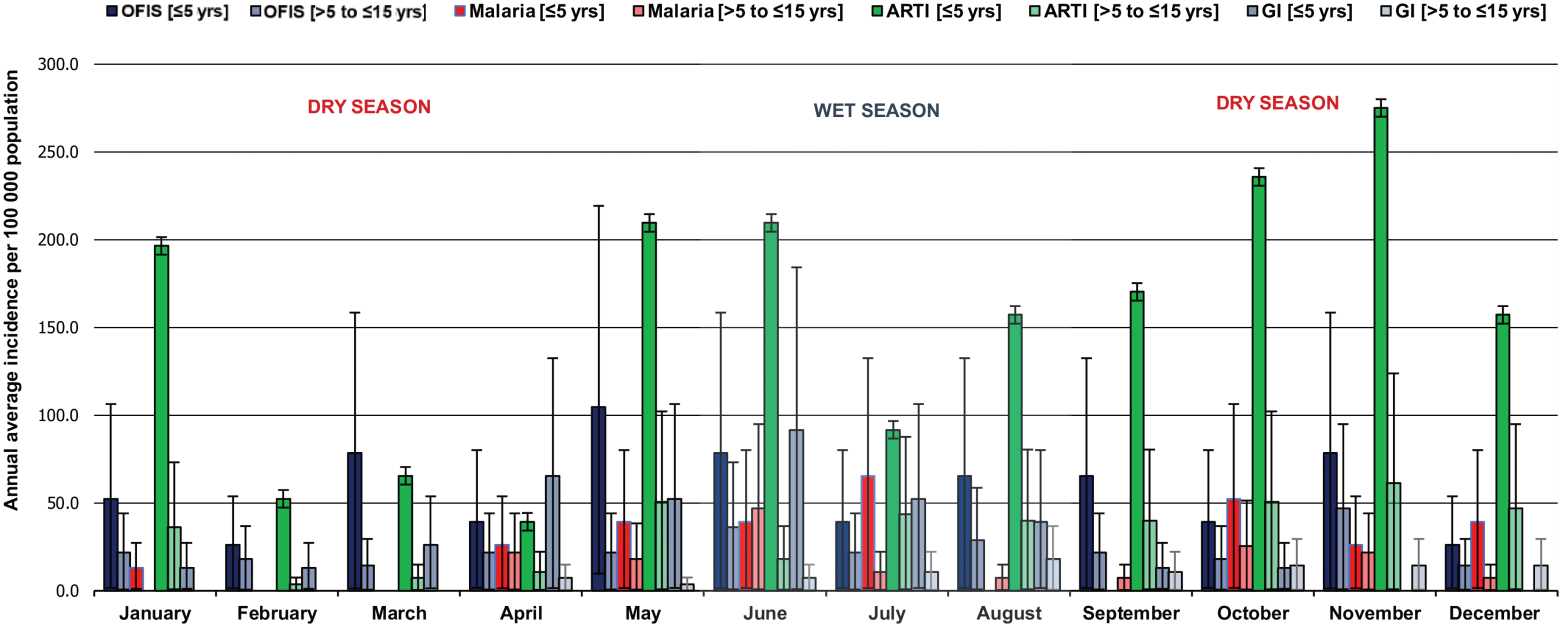
Annual average incidence of infections and syndromes by season, Butajira, Ethiopia, January 2012 to January 2014. The bar chart shows incidence per 100 000 population including the whiskers, which indicate the upper and lower 95% confidence intervals stratified by month and age group. The light red boxes indicate the months of the dry season, and the light blue boxes indicate the months of the wet season in Ethiopia. Abbreviations: ARTI, acute respiratory tract infection; GI, gastrointestinal infection; OFIS, other febrile infections and syndromes.

By applying the case classification scheme, the annual number of new cases per 100 000 population among 17 634 children aged ≤5 years at risk was 301.3 (95% CI, 269.2–337.2) for malaria, 1860.1 (95% CI, 1778.0–1946.0) for ARTI, 379.9 (95% CI, 343.6–420.0) for GI, and 694.3 (95% CI, 644.7–747.7) for OFIS and was generally higher than among 13 817 children aged >5 to ≤15 years at risk. Similarly, irrespective of the disease classified, the monthly incidence rates were higher among patients aged ≤5 years than >5 to ≤15 years (Figure 2). With respect to seasonality, we observed high incidences in children aged ≤5 years for malaria during April–July and October–December; for ARTI throughout the year, except for February–April; for GI during April–August; and for OFIS throughout the year, except for February and December (Figure 2).

**Figure 3.**
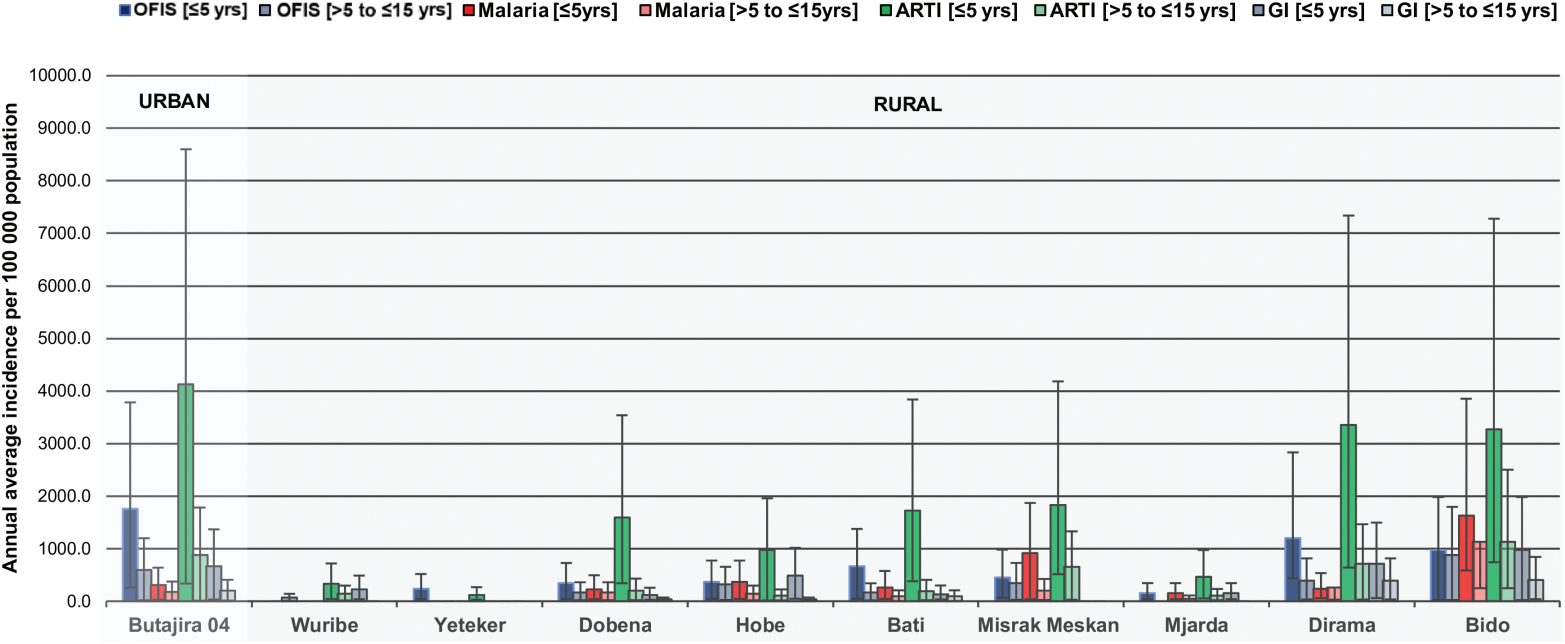
Annual average incidence of infections and syndromes by administrative stratum, Butajira, Ethiopia, January 2012 to January 2014. The bar chart shows incidence per 100 000 population including the whiskers, which indicate the upper and lower 95% confidence intervals stratified by kebele and age group. The light gray boxes indicate the urban kebele and the darker gray boxes indicate the rural kebele of the study site. Abbreviations: ARTI, acute respiratory tract infection; GI, gastrointestinal infection; OFIS, other febrile infections and syndromes.

With respect to spatial occurrence, we found elevated incidence rates of 206.7 (95% CI, 180.4–236.8) for malaria, 1576.6 (95% CI, 1501.0–1656.0) for ARTI, 336.0 (95% CI, 302.0–373.8) for GI, and 840.0 (95% CI, 785.3–898.5) for OFIS in urban participants compared with lower rates for malaria (192.5; 95% CI, 167.2–221.6), ARTI (483.1; 95% CI, 441.9–527.9), GI (112.6; 95% CI, 93.6–135.4), and OFIS (243.0; 95% CI, 214.3–275.5) among rural participants (Figure 3). Similar to annual and monthly data, incidence rates by kebele were generally higher in children aged ≤5 years than in older children. Children who resided in northwestern kebeles, that is, Butajira 04, Misrak Meskan, Dirama, and Bido (Figure 1), were primarily affected by acute febrile illness irrespective of age and disease classification.

### Predictors for Acute Febrile Illness

Multivariate logistic regression revealed a 0.46 times (95% CI, 0.25–0.85; *P* = .013) lower and a 1.79 times (95% CI, 1.20–2.59; *P* < .001) higher odds for malaria and ARTI in children aged ≤5 years than in those aged >5 to ≤15 (Table 3). With respect to seasonality, participants recruited during the dry season had a 0.42 times (95% CI, 0.22–0.78; *P* = .006) lower and a 1.98 times (95% CI, 1.37–2.88; *P* < .001) higher odds for GI and ARTI than those enrolled during the wet season. Residing in urban Butajira was associated with a 0.44 times (95% CI, 0.23–0.84; *P* = .011) lower odds for malaria than rural Butajira. Among children who presented with mild and moderate fever, the odds for malaria were 0.07 times (95% CI, 0.03–0.16; *P* < .001) and 0.12 times (95% CI, 0.05–0.27; *P* < .001) lower than in children who presented with high fever. However, participants with mild and moderate fever had a 3.20 times (95% CI, 1.43–7.17; *P* = .005) and 2.45 times (95% CI, 1.05–5.73; *P* = .039) higher odds for ARTI as well as a 3.10 times (95% CI, 1.04–9.27; *P* = .042) and 3.73 times (95% CI, 1.21–11.54; *P* = .022) higher odds for OFIS than participants with high fever.

**Table 3.** Multivariate Regression Analyses, Butajira, Ethiopia January 2012 to January 2014

		Malaria				Gastrointestinal Infection				Acute Respiratory Tract Infection				Other Febrile Infections and Syndromes			
	Variable	n = 69 (%)	OR	95% CI	*P* Value	n = 57 (%)	OR	95% CI	*P* Value	n = 25 (%)	OR	95% CI	*P* Value	n = 132 (%)	OR	95% CI	*P* Value
Sex	Male	42 (60.9)	1.28	0.72–2.29	.403	32 (56.1)	1.17	0.66–2.07	.580	135 (52.9)	0.82	0.57–1.18	.295	60 (45.5)	1.05	0.69–1.58	.833
	Female	27 (39.1)	1.00	…	…	25 (43.9)	1.00	…	…	120 (47.1)	1.00	…	…	72 (54.4)	1.00	…	…
Age, y	≤5	23 (33.3)	0.46	0.25–0.85	.013	29 (50.9)	1.07	0.61–1.88	.822	142 (55.7)	1.79	1.20–2.59	<.001	53 (40.2)	0.68	0.45–1.04	.073
	>5 to ≤15	46 (66.7)	1.00	…	…	28 (49.1)	1.00	…	…	113 (44.3)	1.00	…	…	79 (59.8)	1.00	…	…
Season^a^	Dry	25 (36.2)	0.62	0.34–1.13	.122	15 (26.3)	0.42	0.22–0.78	.006	128 (50.2)	1.98	1.37–2.88	<.001	51 (38.6)	0.79	0.52–1.21	.283
	Wet	44 (63.8)	1.00	…	…	42 (73.7)	1.00	…	…	127 (49.8)	1.00	…	…	81 (61.4)	1.00	…	…
Setting^b^	Urban	16 (23.2)	0.44	0.23–0.84	.011	26 (45.6)	0.89	0.51–1.60	.717	122 (47.8)	1.16	0.79–1.68	.443	65 (49.2)	1.29	0.86–1.96	.221
	Rural	53 (76.8)	1.00	…	…	31 (54.4)	1.00	…	…	133 (52.2)	1.00	…	…	67 (50.8)	1.00	…	…
Fever^c^	Mild	26 (37.7)	0.07	0.03–0.16	<.001	40 (70.2)	2.52	0.57–11.16	.225	185 (72.5)	3.20	1.43–7.17	.005	89 (67.4)	3.10	1.04–9.27	.042
	Moderate	21 (30.4)	0.12	0.05–0.27	<.001	15 (26.3)	1.98	0.42–9.26	.385	61 (23.9)	2.45	1.05–5.73	.039	39 (29.5)	3.73	1.21–11.54	.022
	High	22 (31.9)	1.00	…	…	2 (3.4)	1.00	…	…	9 (3.5)	1.00	…	…	4 (3.0)	1.00	…	…

Data derived from applying case classification schemes and sex, age, season, setting, and fever type adjusted for pretreatment with antimalarial, antibacterial, and analgesic drugs.

Abbreviations: CI, confidence interval; OR, odds ratio.

^a^Dry season, October–May; wet season, June–September.

^b^Urban, Butajira 04; rural, remaining kebeles.

^c^Body temperature measurement, tympanic.

## DISCUSSION

We investigated the incidence and etiological agents of acute febrile illness among children aged ≤15 years recruited as part of the TSAP in Butajira, south–central Ethiopia using a combination of prospectively collected surveillance data and a retrospectively developed case classification scheme.

We rarely observed stunting, wasting, and underweight, which are signs of severely ill or prolonged chronically diseased children and a likely predictor of mortality. We did not measure the level of dehydration, which is a limitation. However, RBC counts and associated parameters including hemoglobin and hematocrit, both indicative of dehydration if lowered, were within the normal range in most children. We diagnosed lymphocytopenia and neutrophilia in nearly half of children aged ≤5 years and a quarter of children >5 to ≤15 years, respectively. Lowered MCHC, indicative of anemia due to iron deficiency as observed during hemolysis and parasitic infections such as soil-transmitted helminthiases, was found in nearly half of children aged ≤5 years and one-third of children >5 to ≤15 years [30]. A further limitation is that eosinophil counts, indicative of allergic reactions and parasite, entero-parasite, and viral infections; basophil counts, suggestive for inflammatory and allergic reactions; and parasite, ectoparasite infections, and monocyte counts, indicative of severe infections and sepsis, were lacking. Nearly all children presented as outpatients with mild transient fever; we rarely saw severely ill children who required hospitalization. We identified a low mean severity score, though its meaningfulness may be limited since the variable values included were collected only once at study enrollment [29].

We detected 13.5% malaria positivity despite the introduction of intensive control measures since 2005 and Butajira being at a high elevation of >2100 m above sea level [8, 30, 31]. Interestingly, Woyessa et al reported an overall prevalence of <1% from 2008 to 2010 [10]. Malaria infections in our study peaked during April–July and October–December, which is similar to previous findings; Tesfaye et al found 4.4% malaria prevalence during October–December, and Woyessa and colleagues observed high malaria occurrence during October–December [10, 32]. Similar to other malaria research conducted in Ethiopia since the early 20th Century, *P. vivax* and *P. falciparum* were the predominant species and presented with high parasitemia [8, 32–34].

We found 1.6% blood culture positivity for bacterial and fungal pathogens and 10.5% contamination; 85.8% of cultures did not yield any growth. The proportion of contamination was high and could have had an impact on the clinical interpretation and detection of pathogenic agents [12]. Heterogeneity in blood culture sensitivity of 40% to 87% coupled with limited diagnostic capacity in detecting parasitic and viral pathogens could have affected the low detection rate [35]. Blood culture positivity, depending on the duration and range of symptoms and a higher disease severity and thus a likely higher bacteremia, has been discussed [35, 36]. We could show that appropriate blood volumes were added to the bottles according to the manufacturer's instructions by observing the syringe graduation during inoculation, though higher blood volumes could likely have resulted in a higher detection rate. Interestingly, Antillon et al reported 51% and 65% increases in blood culture sensitivity for 2-mL and 10-mL specimens, respectively; sensitivity increased by 3% for each additional milliliter of blood cultured [35]. The mean elapsed time from blood collection to incubation and the mean incubation period until positivity or negativity did not reveal anomalies. Antimicrobial pretreatment could have had an impact on the culture findings seen by Antillon et al and Źródłowski et al who found a decrease in positivity following antibiotherapy [35, 37]. However, data on pretreatment, though rarely reported in our study, have to be interpreted with caution. Nevertheless, inappropriate use of antimicrobials; high exposure, in particular among children; over-the-counter self-medication without medical prescription; and absence of antimicrobial stewardship are reported from Ethiopia [38]. A weakness of our study is that additional variables to better assess blood culture performance, such as the collection side and the temperature and time needed to transport inoculated bottles to the processing laboratory, were poorly recorded.

The bacterial pathogens detected by blood culture could be considered a reflection of the incidence rates derived from applying the case classification scheme and revealed that respiratory and gastrointestinal infections were common, which appears to be consistent with findings from previous research [39]. Surprisingly, caretakers did not consider signs such as infections of the uvula and/or the tonsils, fast breathing, and chest indrawing as sufficiently important to seek medical attention for their sick children [39]. Shamebo et al identified diarrheal diseases and acute respiratory infections as the leading causes of mortality in infants and young children [40]. Likewise, Fantahun et al and Byass et al reported respiratory tract and diarrheal infections in addition to perinatal complications, malaria, measles, malnutrition, and meningitis as the main reasons of death in children aged <5 years in Ethiopia [41, 42]. Despite our findings being consistent with those from other researchers, the incidence rates presented here hold the possibility of bias due to misclassification and should therefore be considered with caution.

## CONCLUSIONS

Children enrolled presented mainly as outpatients with mild transient fever and low disease severity and rarely required hospital admission. We could confirm the etiologies of febrile illnesses due to bacterial and fungal pathogens and malaria parasites for 1.6% and 13.5% children from Butajira, south–central Ethiopia. Incidence rates could be considered a reflection of culture findings and previous research as they revealed that respiratory and gastrointestinal infections were common, especially among young children. The developed case classification scheme could be regarded as a surrogate of fever-causing etiologies in light of the sparse blood culture outcomes. However, when interpreting our findings, the impact of numerous disease control and prevention programs that are ongoing in Ethiopia to improve health education, access to safe drinking water and sanitation, and healthcare infrastructure should also be considered. Settings such as Butajira require a broad range of diagnostic tools beyond blood culture to assess the definite causes of fever. Once the etiological agents are clearly defined, healthcare personnel and decision makers should develop revised policies and frameworks for the management and prevention of acute febrile illness.

## Supplementary Data

Supplementary materials are available at *Clinical Infectious Diseases* online. Consisting of data provided by the authors to benefit the reader, the posted materials are not copyedited and are the sole responsibility of the authors, so questions or comments should be addressed to the corresponding author.

ciz620_suppl_Supplemental_Figure_1

ciz620_suppl_Supplemental_Figure_2

ciz620_suppl_Supplemental_Table_1

ciz620_suppl_Supplemental_Table_2

ciz620_suppl_Supplemental_Table_3

ciz620_suppl_Supplemental_Figure_Legends

## Notes

***Author contributions.*** M. T., B. Y., M. Y., and T. B. conduct the Typhoid Fever Surveillance in Africa Program in Butajira, including the recruitment of patients and data collection under the scientific leadership of A. A. and F. M. and with scientific and operational support of U. P., L. M. C. E., F. K., S. E. P., J. I., H. J., and S. Y. K. G. D. P. and J. K. P. provided data management support throughout the study. M. D., M. T., and U. P. conceptualized and drafted the manuscript with scientific support of J. K., J. H. K., A. A., S. B., F. M., and W. S. S. All authors read and approved the final manuscript.

***Acknowledgments.*** The authors are very grateful to the residents in Butajira, Ethiopia, for their participation in this study and thank the healthcare personnel at the centers and hospitals in Butajira for their exceptional efforts and contributions, which made this research possible. The Armauer Hansen Research Institute acknowledges financial support from the Norwegian and Swedish International Development Agencies, Norad and Sida. The authors thank Marianne Holm for her scientific contribution to this manuscript.

***Financial support.*** This research was funded by the Bill & Melinda Gates Foundation (OPP1127988). The International Vaccine Institute acknowledges its donors including the Republic of Korea and the Swedish International Development Cooperation Agency. This publication was made possible through a grant from the Bill & Melinda Gates Foundation (OPP1201031).

***Supplement sponsorship.*** This article was published as part of the supplement “Severe Typhoid Fever in Africa (SETA) Program” sponsored by the International Vaccine Institute.

***Potential conflicts of interest.*** J. K. reports payments from Takeda outside the submitted work. All other authors report no potential conflicts. All authors have submitted the ICMJE Form for Disclosure of Potential Conflicts of Interest. Conflicts that the editors consider relevant to the content of the manuscript have been disclosed.
