# Supplementary figures and images for: Acute Febrile Illness Among Children in Butajira, South–Central Ethiopia During the Typhoid Fever Surveillance in Africa Program
Source: Clin Infect Dis. 2019 Oct 30;69(Suppl 6):S483–91. doi: 10.1093/cid/ciz620 (PMC6821253; doi:10.1093/cid/ciz620)

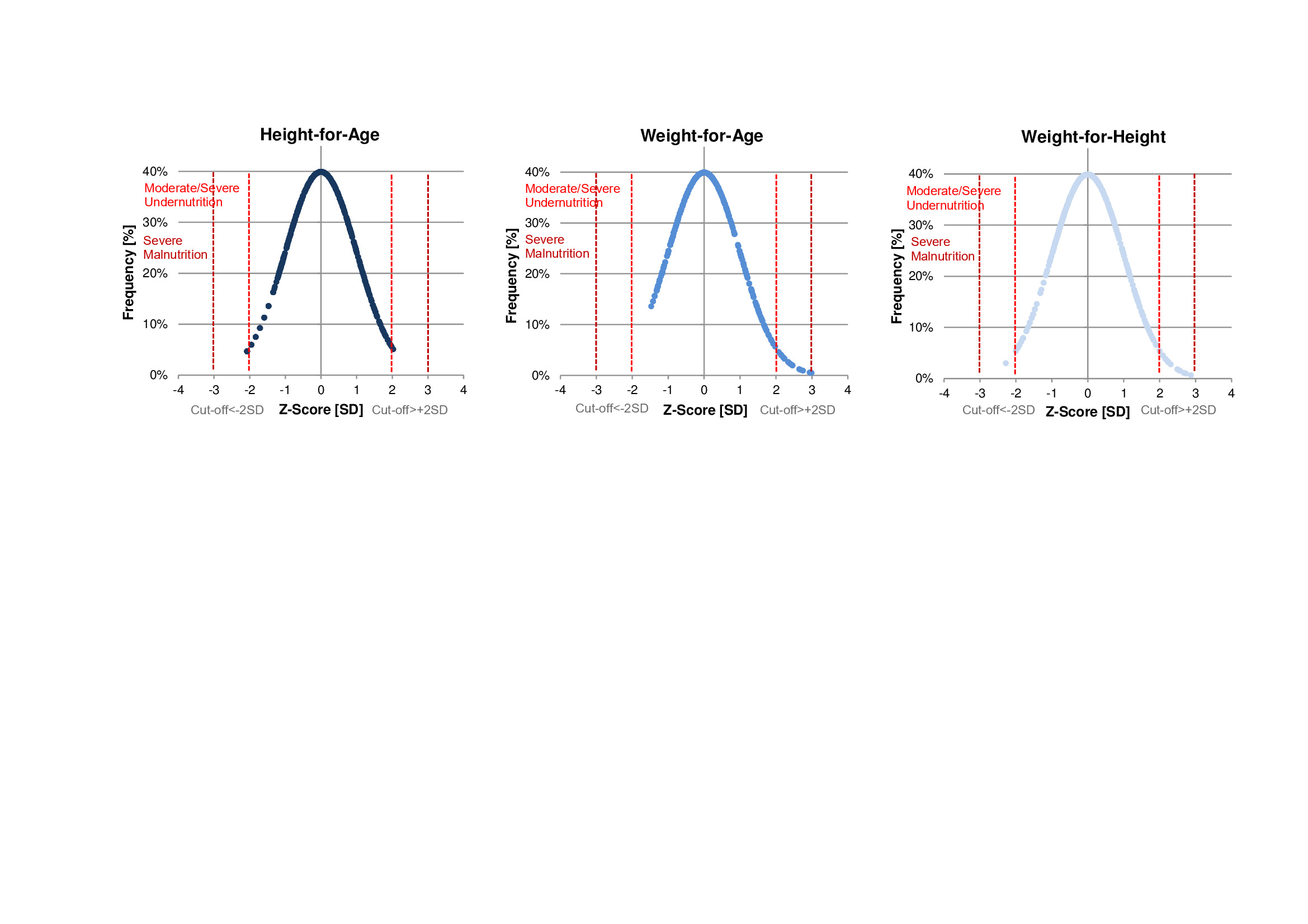

Supplement: ciz620_suppl_Supplemental_Figure_1 [file ciz620_suppl_supplemental_figure_1.jpeg]

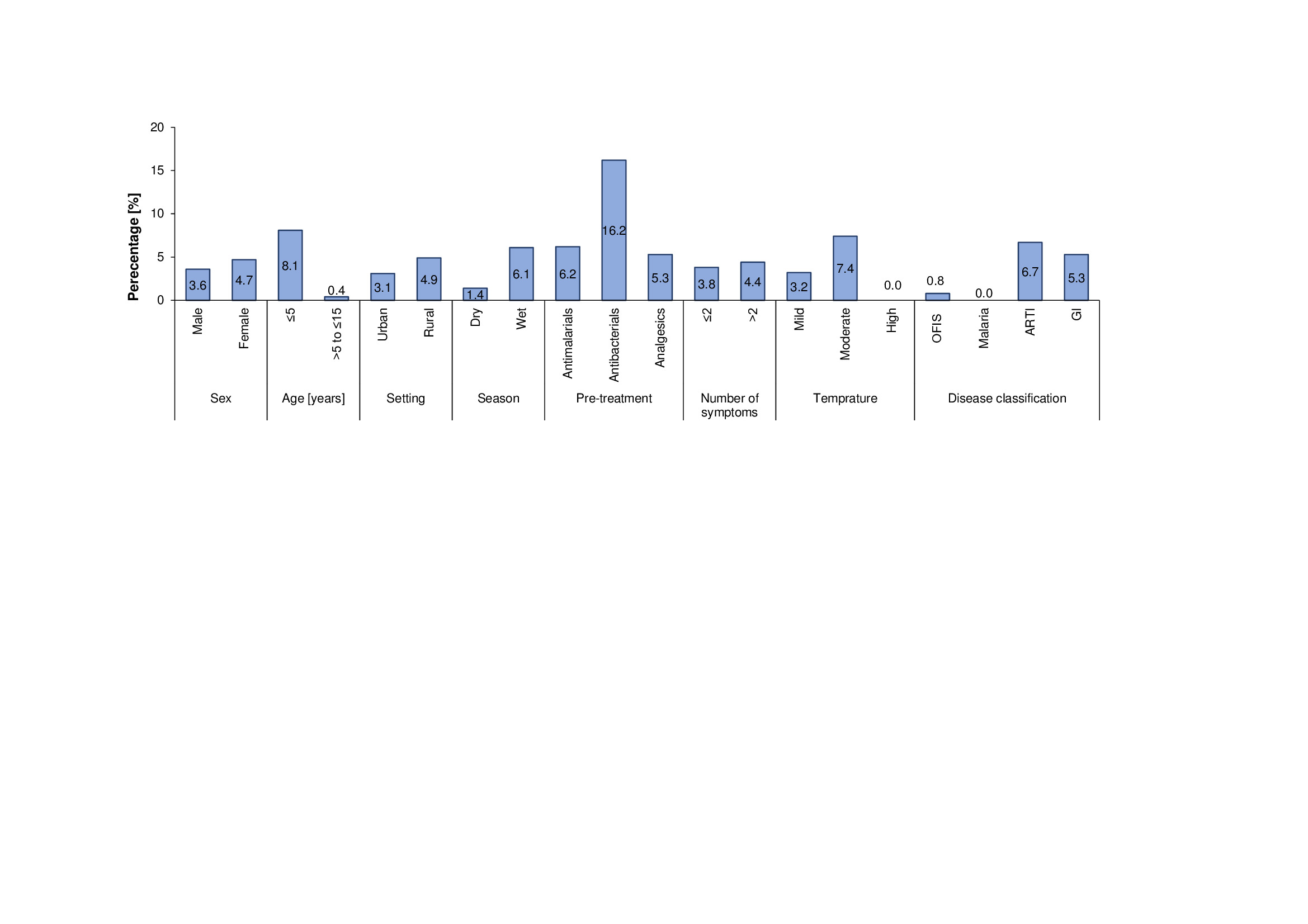

Supplement: ciz620_suppl_Supplemental_Figure_2 [file ciz620_suppl_supplemental_figure_2.jpeg]
