# Supplementary material for: Acute Febrile Illness Among Children in Butajira, South–Central Ethiopia During the Typhoid Fever Surveillance in Africa Program
Source: Clin Infect Dis. 2019 Oct 30;69(Suppl 6):S483–91. doi: 10.1093/cid/ciz620 (PMC6821253; doi:10.1093/cid/ciz620)
Supplement: ciz620_suppl_Supplemental_Table_1 [file ciz620_suppl_supplemental_table_1.docx]

**Table 3.** Clinical characteristics of enrolled children, Butajira, Ethiopia January 2012 to January 2014 (*online supplementary*) .

| **Characteristics** | | **All children**  n=513  (%) | **Sex** | | **Age group** [years] | | **Setting** | | **Season** | |
| --- | --- | --- | --- | --- | --- | --- | --- | --- | --- | --- |
|  |  |  | Male  n=281 (%) | Female  n=232  (%) | ≤5  n=247  (%) | ˃5 to ≤15 n=266  (%) | Urban n=229 (%) | Rural n=284  (%) | Dry n=219  (%) | Wet n=294  (%) |
| **Symptom** | Diarrhea | 115 (22.4) | 62 (53.9) | 53 (46.1) | 68 (59.1) | 47 (40.9) | 62 (53.9) | 53 (46.1) | 39 (33.9) | 76 (66.1) |
|  | Headache | 295 (57.5) | 167 (56.6) | 128 (43.4) | 87 (29.5) | 208 (70.5) | 115 (39.0) | 180 (61.0) | 136 (46.1) | 159 (53.9) |
|  | Constipation | 14 (2.7) | 8 (57.1) | 6 (42.9) | 2 (14.3) | 12 (85.7) | 7 (50.0) | 7 (50.0) | 4 (28.6) | 10 (56.9) |
|  | Sore throat | 62 (12.1) | 33 (53.2) | 29 (46.8) | 25 (40.3) | 37 (59.7) | 33 (53.2) | 29 (46.8) | 23 (37.1) | 39 (62.9) |
|  | Rash | 6 (1.2) | 1 (16.7) | 5 (83.3) | 4 (66.7) | 2 (33.3) | 3 (50.0) | 3 (50.0) | 2 (33.3) | 4 (66.7) |
|  | Cough | 252 (49.1) | 129 (51.2) | 123 (48.8) | 137 (54.4) | 115 (45.6) | 104 (41.3) | 148 (58.7) | 123 (48.8) | 129 (51.2) |
|  | Vomiting | 173 (33.7) | 100 (57.8) | 73 (42.2) | 88 (50.9) | 85 (49.1) | 76 (43.9) | 97 (56.1) | 69 (39.9) | 104 (60.1) |
|  | Abdominal pain | 69 (13.5) | 37 (53.6) | 32 (46.4) | 27 (39.7) | 41 (60.3) | 24 (34.8) | 45 (65.2) | 25 (36.2) | 44 (63.8) |
|  | Others | 68 (13.3) | 42 (61.8)) | 26 (38.2) | 27 (39.7) | 41 (60.3) | 34 (50.0) | 34 (50.0) | 21 (30.9) | 47 (69.1) |
| **Body temperature** | Mild [≤38.9°C] | 340 (66.3) | 176 (51.8) | 164 (48.2) | 172 (50.6) | 168 (49.4) | 176 (51.8) | 164 (48.2) | 153 (45.0) | 187 (55.0) |
|  | Moderate [≥39.0 ≤39.9°C] | 136 (26.5) | 82 (60.3) | 54 (39.7) | 57 (41.9) | 79 (58.1) | 45 (33.1) | 91 (66.9) | 51 (37.5) | 85 (62.5) |
|  | High [≥40.0°C] | 37 (17.2) | 23 (62.2) | 14 (37.8) | 18 (48.6) | 19 (51.4) | 8 (21.6) | 29 (78.4) | 15 (40.5) | 22 (59.5) |
|  | Fever continua | 219 (42.7) | 126 (57.8) | 93 (42.2) | 112 (51.1) | 107 (49.1) | 80 (36.7) | 139 (63.3) | 95 (43.6) | 124 (56.4) |
|  | Intermittent fever | 294 (57.3) | 155 (52.7) | 139 (47.3) | 135 (45.9) | 159 (54.1) | 149 (50.7) | 145 (49.3) | 124 (42.2) | 170 (57.8) |
|  | Fever ≤3days | 469 (91.4) | 261 (55.7) | 208 (44.3) | 224 (47.8) | 245 (52.2) | 213 (45.4) | 256 (54.6) | 203 (43.3) | 266 (56.7) |
|  | Fever >3days | 44 (8.6) | 20 (45.5) | 24 (54.5) | 23 (52.3) | 21 (47.7) | 16 (36.4) | 28 (63.6) | 16 (36.4) | 28 (63.6) |
| **Healthcare facility** | Butajira hospital | 182 (35.5) | 94 (51.6) | 88 (48.4) | 122 (67.0) | 60 (33.0) | 136 (74.7) | 46 (25.3) | 66 (36.3) | 116 (63.7) |
|  | Butajira health center | 125 (24.4) | 69 (55.2) | 56 (44.8) | 33 (26.4) | 92 (73.6) | 83 (66.4) | 42 (33.6) | 53 (42.4) | 72 (57.6) |
|  | Shershera Bido health center | 97 (18.9) | 64 (66.0) | 33 (34.0) | 33 (34.0) | 64 (66.0) | 3 (3.1) | 94 (96.9) | 43 (44.3) | 54 (55.7) |
|  | Enseno health center | 109 (21.2) | 54 (49.5) | 55 (50.5) | 59 (54.1) | 50 (45.9) | 7 (6.4) | 102 (93.6) | 57 (52.3) | 52 (47.7) |
| **Contact with febrile person** | | 40 (7.8) | 22 (55.0) | 18 (45.0) | 16 (40.0) | 24 (60.0) | 13 (32.5) | 27 (67.5) | 21 (52.5) | 19 (47.5) |
| **Travel outside the study area** | | 13 (2.5) | 7 (53.8) | 6 (46.2) | 7 (53.8) | 6 (46.2) | 1 (7.7) | 12 (92.3) | 4 (30.8) | 9 (69.2) |

Setting: urban: Butajira 04, rural: remaining Kebeles; Season: dry season: October-May, wet season: June-September; Body temperature measurement: tympanic.
