# Supplementary material for: Acute Febrile Illness Among Children in Butajira, South–Central Ethiopia During the Typhoid Fever Surveillance in Africa Program
Source: Clin Infect Dis. 2019 Oct 30;69(Suppl 6):S483–91. doi: 10.1093/cid/ciz620 (PMC6821253; doi:10.1093/cid/ciz620)
Supplement: ciz620_suppl_Supplemental_Table_2 [file ciz620_suppl_supplemental_table_2.docx]

**Table 4.** Baseline characteristics of enrolled children, Butajira, Ethiopia January 2012 to January 2014 (*online supplementary*).

| **Characteristics** | | **All children**  n=513  (%~~)~~ | **Sex** | | **Age group** [years] | | **Setting** | | **Season** | |
| --- | --- | --- | --- | --- | --- | --- | --- | --- | --- | --- |
|  |  |  | Male  n=281  (%) | Female  n=232  (%) | ≤5  n=247  (%) | ˃5 to ≤15  n=266  (%) | Urban n=229  (%) | Rural  n=284  (%) | Dry  n=219  (%) | Wet  n=294  (%) |
| **Case classification** | Malaria | 69 (13.5) | 42 (60.9) | 27 (39.1) | 23 (33.3) | 46 (66.7) | 16 (23.2) | 53 (76.8) | 25 (36.2) | 44 (63.8) |
|  | ARTI | 255 (49.7) | 135 (52.9) | 120 (47.1) | 142 (55.7) | 113 (44.3) | 122 (47.8) | 133 (52.2) | 128 (50.2) | 127 (49.8) |
|  | GI | 57 (11.1) | 32 (56.1) | 25 (43.9) | 29 (50.9) | 28 (49.1) | 26 (45.6) | 31 (54.4) | 15 (26.3) | 42 (73.7) |
|  | OFIS | 132 (25.7) | 72 (54.5) | 60 (45.5) | 53 (40.2) | 79 (59.8) | 65 (49.2) | 67 (50.8) | 51 (38.6) | 81 (61.4) |

GI: Gastrointestinal Infection, OFIS: Other febrile infections and syndromes, ARTI: Acute Respiratory Tract Infection; Setting: urban: Butajira 04, rural: remaining Kebeles; Season: dry season: October-May, wet season: June-September.
