# Supplementary material for: Acute Febrile Illness Among Children in Butajira, South–Central Ethiopia During the Typhoid Fever Surveillance in Africa Program
Source: Clin Infect Dis. 2019 Oct 30;69(Suppl 6):S483–91. doi: 10.1093/cid/ciz620 (PMC6821253; doi:10.1093/cid/ciz620)
Supplement: ciz620_suppl_Supplemental_Table_3 [file ciz620_suppl_supplemental_table_3.docx]

**Table 5.** Treatment of recruited children, Butajira, Ethiopia January 2012 to January 2014 (*online supplementary*) .

| **Characteristics** | | **All children**  n=513  (%) | **Sex** | | **Age group** [years] | | **Setting** | | | **Season** |
| --- | --- | --- | --- | --- | --- | --- | --- | --- | --- | --- |
|  |  |  | Male  n=281  (%) | Female  n=232  (%) | ≤5  n=247  (%) | ˃5 to ≤15  n=266  (%) | Urban  n=229  (%) | Rural  n=284  (%) | Dry  n=219  (%) | Wet  n=294  (%) |
| **Pre-treatment** (self-reported) | Antimalarials | 16 (3.1) | 7 (43.8) | 9 (56.2) | 7 (43.8) | 9 (56.2) | 5 (31.2) | 11 (68.8) | 7 (43.8) | 9 (56.2) |
|  | Antibacterials | 37 (7.2) | 16 (43.2) | 21 (56.8) | 29 (78.4) | 8 (21.6) | 23 (62.2) | 14 (37.8) | 15 (40.5) | 22 (59.5) |
|  | Analgesics | 57 (11.1) | 31 (54.4) | 26 (45.6) | 29 (50.9) | 28 (49.1) | 24 (42.1) | 33 (57.9) | 25 (43.9) | 32 (56.1) |
| **Antibacterial prescribed** (at discharge) | Ampicillin | 6 (1.2) | 4 (66.7) | 2 (33.3) | 1 (16.7) | 5 (83.3) | 4 (66.7) | 2 (33.3) | 2 (33.3) | 4 (66.7) |
|  | Ciprofloxacin | 47 (9.2) | 32 (68.1) | 15 (31.9) | 10 (21.3) | 37 (78.7) | 7 (14.9) | 40 (85.1) | 26 (55.3) | 21 (44.7) |
|  | Gentamicin | 2 (0.4) | 1 (50.0) | 1 (50.0) | 2 (100.0) | 0 (0.0) | 1 (50.0) | 1 (50.0) | 0 (0.0) | 2 (100.0) |
|  | Chloramphenicol | 32 (6.2) | 13 (40.6) | 19 (59.4) | 11 (34.4) | 21 (65.6) | 15 (46.9) | 17 (53.1) | 17 (53.1) | 15 (46.9) |
|  | Co-trimoxazole | 68 (13.3) | 38 (55.9) | 30 (44.1) | 47 (69.1) | 21 (30.9) | 30 (44.1) | 38 (55.9) | 25 (36.8) | 43 (63.2) |
|  | Ceftriaxone | 1 (0.2) | 0 (0.0) | 1 (100.0) | 1 (100.0) | 0 (0.0) | 0 (0.0) | 1 (100.0) | 0 (0.0) | 1 (100.0) |
|  | Tetracycline | 2 (0.4) | 2 (100.0) | 0 (0.0) | 0 (0.0) | 2 (100.0) | 1 (50.0) | 1 (50.0) | 2 (100.0) | 0 (0.0) |
|  | Amoxicillin | 145 (28.3) | 75 (51.7) | 70 (48.3) | 73 (50.3) | 72 (49.7) | 92 (63.4) | 53 (36.6) | 61 (42.1) | 84 (57.9) |
|  | Other | 301 (58.7) | 164 (54.5) | 137 (45.5) | 158 (52.5) | 143 (47.5) | 112 (37.2) | 189 (62.8) | 136 (45.2) | 165 (54.8) |

Setting: urban: Butajira 04, rural: remaining Kebeles; Season: dry season: October-May, wet season: June-September.
