# Supplementary material for: Acute Febrile Illness Among Children in Butajira, South–Central Ethiopia During the Typhoid Fever Surveillance in Africa Program
Source: Clin Infect Dis. 2019 Oct 30;69(Suppl 6):S483–91. doi: 10.1093/cid/ciz620 (PMC6821253; doi:10.1093/cid/ciz620)
Supplement: ciz620_suppl_Supplemental_Figure_Legends [file ciz620_suppl_supplemental_figure_legends.docx]

**Figure legends of online supplementary figures**

**Supplement Figure 1.** Physical development metrics in recruited children, Butajira, Ethiopia January 2012 to January 2014.

Z-scores for height-for-age, weight-for-age and weight-for-height; SD: standard deviation.

**Supplemental Figure 2.** Characteristics of admitted children, Butajira, Ethiopia January 2012 to January 2014.

Setting: Urban: Butajira 04, Rural: remaining Kebeles; Season: Dry season: October-May, Wet season: June-September; Pre-treatment: Self-reported; Body temperature measurement: Mild: ≤38.9°C, Moderate: ≥39.0°C to ≤39.9°C, High: ≥40.0°C; OFIS: Other febrile infections and syndromes, ARTI: Acute Respiratory Tract Infection, GI: Gastrointestinal Infection
